# Selenium and sulindac are synergistic to inhibit intestinal tumorigenesis in *Apc/p21* mice

**DOI:** 10.1186/1756-8722-6-8

**Published:** 2013-01-17

**Authors:** Xiuli Bi, Nicole Pohl, Huali Dong, Wancai Yang

**Affiliations:** 1 School of Life Sciences, Liaoning University, Shenyang, 110036, China; 2 Department of Pathology, University of Illinois at Chicago, Chicago, IL, 60612, USA; 3 Cancer Center, University of Illinois at Chicago, Chicago, IL, 60612, USA; 4 Department of Pathology, Xinxiang Medical University, 601 East Jinsui Dadao, Xinxiang, Henan, 453003, China

**Keywords:** Selenium, Sulindac, Cancer prevention, Methylation, Wnt/β-catenin

## Abstract

**Background:**

Both selenium and non-steroidal anti-inflammatory drug (NSAID) sulindac are effective in cancer prevention, but their effects are affected by several factors including epigenetic alterations and gene expression. The current study was designed to determine the effects of the combination of selenium and sulindac on tumor inhibition and the underlying mechanisms.

**Results:**

We fed the intestinal tumor model Apc/p21 mice with selenium- and sulindac-supplemented diet for 24 weeks, and found that the combination of selenium and sulindac significantly inhibited intestinal tumorigenesis, in terms of reducing tumor incidence by 52% and tumor multiplicities by 80% (p<0.01). Mechanistic studies revealed that the combination of selenium and sulindac led to the significant induction of the expression of p27 and p53 and JNK1 phosphorylation, and led to the suppression of β-catenin and its downstream targets. Impressively, the data also showed that demythelation on p21 promoter was associated with tumor inhibition by the combination of selenium and sulindac.

**Conclusions:**

The selenium is synergistic with sulindac to exert maximal effects on tumor inhibition. This finding provides an important chemopreventive strategy using combination of anti-cancer agents, which has a great impact on cancer prevention and has a promising translational potential.

## Background

Sulindac is a non-steroidal anti-inflammatory drug (NSAID) that has shown to be effective in preventing intestinal tumors in familial adenomatosis polyposis patients that inherit a mutant allele of the adenomatosis polyposis coli (*APC*) gene [1,2], in inhibiting tumor formation in mouse genetic models (ApcMin and Apc1638N+) in which an allele of the homologous mouse *Apc* gene is inactivated by a mutation [3-5], and in inhibiting carcinogen-induced colon tumor formation in rats [6]. Our previously studies have demonstrated that loss of *p21WAF1/Cip*1 gene could enhance intestinal tumorigenesis in the Apc mouse model (i.e. *Apc/p21* mice) [7]. Most interestingly, dietary supplemental sulindac was able to inhibit tumor formation in the *Apc+/−/p21+/+* mice, but not in the mice in which even a single p21 allele was inactivated (i.e. *Apc+/−/p21+/−*) [3], because the remaining wild-type p21 allele is inactivated by hypermethylation of a CpG cluster in its promoter in both the intestinal mucosa as well as in the tumors that form [8]. To determine whether the hypermethylated mouse p21 promoter could be demethylated, and then the response to sulindac on intestinal tumor formation could be resumed, we fed the *Apc/p21* mice with diets supplemented with selenium or combination of selenium and sulindac in present study to determine the intestinal tumor inhibition.

Selenium, an important trace element, is essentially involved in different physiological functions in mammalian and human body. Selenium has significant activity as a chemoprevention agent for cancer. Epidemiological and experimental studies have suggested that intake of dietary selenium is inversely related to overall cancer risk. The effect was most pronounced in gastrointestinal and prostate cancer [9-11]. In addition, *in vivo* studies have demonstrated that dietary selenium supplementation can reduce cancer incidence in animal models of melanoma and cancers of colon, breast, liver, esophagus, head and neck, kidney and lung [10,11]. The anti-cancer effects of selenium have been postulated to link to inhibition of cell proliferation and induction of apoptosis through different signaling pathways, particularly the anti-oxidative and anti-inflammatory effects mediated through the activity of selenoenzymes [12]. While the targets and underlying mechanisms of anti-cancer action by selenium are largely unknown. Recently, our group found that sodium selenite inhibits intestinal carcinogenesis *in vivo and in vitro* through a novel anti-cancer mechanism - activating JNK1 and suppressing β-catenin signaling [13], in addition to the action of selenium of impacting methylation by inhibiting DNA methyltransferase [14,15].

The unique *Apc/p21* mouse model of intestinal tumor was applied in the current student. We found that selenium was synergistic with sulindac and exerted maximum tumor inhibition efficacy through inhibiting p21 promoter methylation, inducing p53, p27 and phosphorylation of JNK1, and suppressing Wnt/β-catenin signaling, although selenium alone showed slight inhibitory effect in the *Apc/p21* mice.

## Results

### Combination of selenium and sulindac significantly decreased intestinal tumorigenesis in *Apc+/−/p21+/−* mice

**Figure 1:**
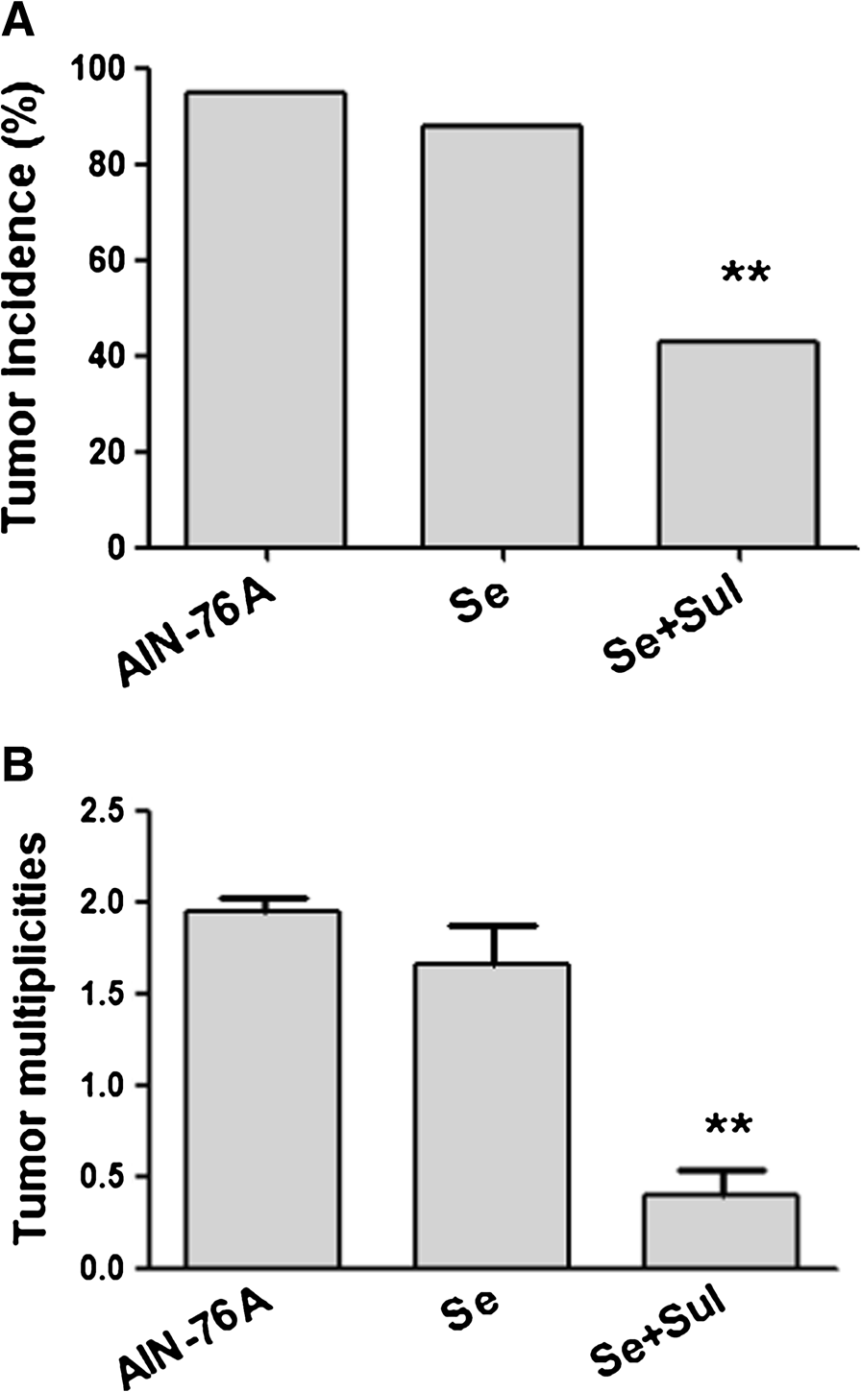
**Combination of selenium and sulindac significantly inhibited intestinal tumor incidence (A) and multiplicities (B) in the *Apc/p21+/−* mice.** The mice were fed AIN-76A, AIN-76A plus selenium or AIN-76A plus selenium and sulindac diet for 24 weeks (p<0.01, compared to the mice fed AIN-76A diet). “Se” stands for AIN-76A+selenium; “*Se+Sul*” stands for AIN-76A+selenium +sulindac.

In consistent with our earlier report [7], loss of p21 increased Apc-initiated intestinal tumorigenesis. Approximately 95% (18/19) of the *Apc+/−/p21+/−* mice spontaneously developed intestinal tumors when they fed with the AIN-76A defined diet, at average multiplicities of 1.95 per mouse (Figure 1). Selenium alone slightly inhibited intestinal tumor formation in the *Apc+/−/p21+/−* mice, tumor incidence decreased to 88% (23/26) and tumor multiplicity decreased to 1.66, the differences were not significant (P> 0.05), in comparison with the mice fed the AIN-76A diet.

Our earlier study also reported that sulindac did not exert tumor inhibition in *Apc+/−/p21+/−* and *Apc+/−/p21−/−* mice although sulindac inhibited tumorigenesis in the *Apc+/−/p21+/+* mice in which both *P21* alleles were wild-type [3]. However, the combination of sulindac and selenium showed significant tumor inhibition in the Apc/p21 mice in the present study. As shown in Figure 1, when the mice were fed the diet supplemented with both sodium selenite and sulindac, intestinal tumor incidence decreased 52% from 95% to 43% (6/14) (Figure 1A) and tumor multiplicities decreased about 80% from 1.95 to 0.4 per mouse (Figure 1B). Compared to the AIN-76A group, the differences of tumor incidence and multiplicity were significant, (p<0.01). These data strongly suggested that selenium be synergistic to sulindac and exert better chemopreventive effects on intestinal tumor formation in the *Apc+/−/p21+/−* mice.

### Intestinal tumor inhibition by selenium and sulindac was associated with suppressing Wnt/β-catenin signaling pathway

**Figure 2:**
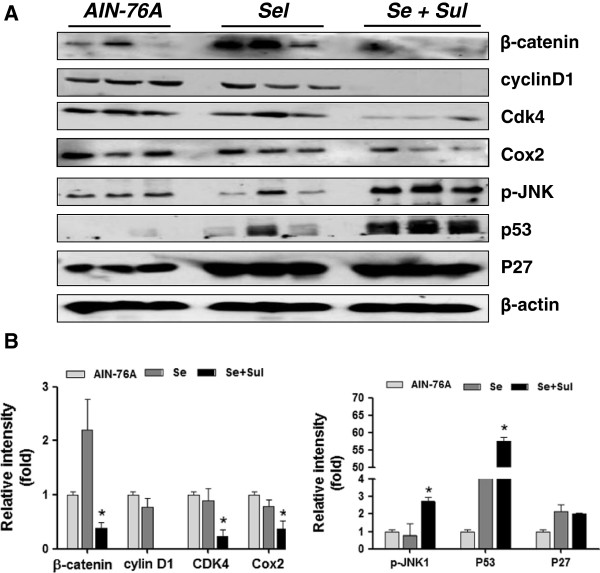
**A. Protein alterations by selenium and sulindac in intestinal epithelial cells from the* Apc+/−/p21+/−* mice, assayed by immunoblotting. B**. Quantification of protein intensity by Quanty One. “*Se*” stands for AIN-76A+selenium; “*Se+Sul*” stands for AIN-76A+selenium +sulindac*.* (p<0.01, compared to the mice fed AIN-76A diet).

To elucidate underlying mechanisms of tumor inhibition by selenium and sulindac, the potential changes of molecules involved in Wnt-β-catenin signaling pathways were determined. As shown in Figure 2, in comparison with the AIN-76A group, combination of selenium and sulindac significantly suppressed the expression of β-catenin, cyclin D1 and cdk4 by 2.6-fold, 100-, and 4.2-fold, respectively. Interestingly, inflammatory marker Cox2 was also decreased by 2.7-fold. In contrast, phosphorylated JNK1 (active form of JNK1) was increased 2.7-fold, p27 was increased 2-fold and p53 was upregulated by 57-fold in the mouse intestinal epithelial cells treated with the combination of selenium and sulindac. However, compared to the AIN-76A control group, the protein levels in the selenium group did not show significant change, which might explain its non-significant effect on tumor inhibition. It is noted that since sulindac alone was not sufficient to inhibit intestinal tumorigenesis in the *Apc/p21+/−* mice [3], and the p21 protein was not inducible in intestinal epithelial cells from the *Apc/p21+/−*[3,8], no further molecular markers were determine in the sulindac alone mouse group in current study.

### Selenium and sulindac synergistically affected mouse p21 promoter methylation

**Figure 3:**
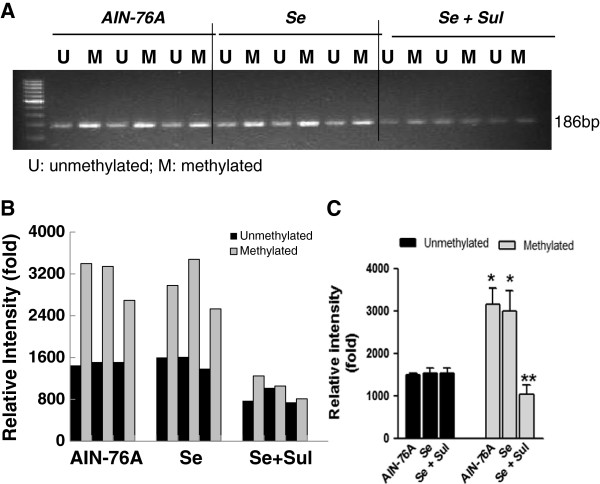
**Combination of selenium and sulindac impacted p21 promoter methylation in intestinal epithelial cells of *Apc/p21+/− *mice (A). “*Se” *stands for AIN-76A+selenium; “*Se+Sul*” stands for AIN-76A+selenium +sulindac. B**. Quantification of the MS-PCR band intensity in individual mouse. **C**, Overall average of the MS-PCR band intensity in each group. In AIN-76A and Se alone group, the methylation was higher than unmethylation (*p<0.05, compared to the unmethylation groups), but in Se + Sul groups, the methylation status was significantly reversed into unmethylation (**p<0.01, compared to the unmethylation groups).

Our previous study has demonstrated that the distal CpG region of mouse promoter of the remaining wild-type p21 allele was hypermethylated in intestinal epithelial cells [8]. To gain further insight into the mechanisms by which selenium could be synergistic with the sulindac to exert tumor inhibition in the *Apc+/−/p21+/−* mice, the impact on p21 promoter methylation status was evaluated using the isolated intestinal epithelial cells from different mouse groups, and the methylation-specific PCR was performed. Consistent with our previous report, mouse p21promoter distal CpG region was hypermethylated in intestinal epithelial cells of the *Apc+/−/p21+/−* mice. Selenium and sulindac synergistically impacted mouse p21 promoter methylation, although selenium alone did not (Figure 3). As one allele of p21 in the *Apc/p21+/−* mice was lost, p21 protein levels were very low and were not induced by sulindac [8] or selenium alone, the induction of p21 protein by the combination of selenium and sulindac was not obvious, and the induction of p21 mRNA was not significant either (data not shown), assayed by immunoblotting and quantitative RT-PCR as described previously [8].

## Discussion

Epigenetic alterations, particularly hypermethylation of CpG islands located in the promoter regions of tumor suppressor genes, contribute to tumor formation. Therefore, developing chemopreventive strategies using the agents targeting methylation will have great translational potential. Using the unique mouse model of intestinal tumors in this study, incorporating with our earlier results [3,4,8], we found that trace element selenium and sulindac synergistically inhibited intestinal tumor formation.

Several lines of evidences have proven that selenium is effective in preventing tumorigenesis. For instance, several randomized controlled trials have demonstrated significant cancer prevention of selenium in colorectal cancer [16-19] and in *Muc2/p21* mouse model of intestinal cancer reported recently by us [13]. However, the selenium and vitamin E clinical trial (SELECT) on prostate cancer prevention was failed and had to be terminated earlier [20], although several factors should be considered. In the current Apc/p21 mouse model, selenium alone showed slight effect on tumor inhibition. It has been demonstrated that anti-cancer action of selenium is through multiple mechanisms. One of the novel mechanisms is to impact methylation. Unlike previous reports, we observed slight tumor inhibitory effect of selenium, and almost no effect on methylation. Sulindac alone has not been reported any demethylation or inhibitory effect on DNA transmethylferase. However, the combination of selenium and sulindac dramatically inhibited tumor formation and significantly affected mouse p21 promoter methylation level. Therefore, this combination of selenium and sulindac could pave a new path for cancer prevention, and had promising translational significance.

Our mechanistic study provided further insight of the combination of selenium and sulindac in cancer prevention, for example, selenium was synergistic with sulindac to suppress Wnt/β-catenin signaling and induce Wnt/β-catenin inhibitors. It is worth to point out that, the combination of selenium and sulindac significantly induced the expression of p27 and p53 and phosphorylation of JNK1. It is well known that abnormally low levels of p27 protein are frequently found in human carcinomas, and these low levels are directly correlated with histological aggressiveness, lymph node metastasis and poor prognosis of esophagus, gastric, breast and colorectal carcinomas [21,22]. Overexpression of p27 and p53 could downregulate β-catenin and its downstream targets, inhibit cell proliferation and promote cell differentiation and apoptosis [23-25]. Most importantly, phosphorylated JNK1 plays a critical role in intestinal cell maturation, e.g. inducing apoptosis and differentiation and inhibiting proliferation [26] through negatively interacting with β-catenin and facilitating β-catenin degradation *in vivo and in vitro*[27,28]. There are a lot of JNK1 stimulating agents, among them, both selenium and sulindac are most effective in activating JNK1 [13,29]. Thus, the combination of selenium and sulindac could exhibit maximum tumor inhibitory effects in current study. These results were different with our previous study in another mouse model of intestinal cancer – *Muc2/p21* mice [13], in which we found that selenium supplemented in the Western-style high risk diet was able to inhibit intestinal tumorigenesis and the selenium alone was likely to induce JNK1 phosphorylation and to inhibit β-catenin and Cox-2. The causes of this discrepancy could be resulted from the differences of mouse model backgrounds and diets. In that study [13], the *Muc2/p21* model was generated from mating *p21−/−* mice with *Muc2* knockout mice, in which *Muc2* gene plays a critical role in causing tumor formation and β-catenin is inactivated [30]; in the current study, the *Apc/p21* mice were generated from mating of *p21−/−* mice with *Apc1638+/−* mice, in which *Apc* mutation plays a causing role on tumor formation because β-catenin is activated [31]. In addition, in the previous study, sodium selenite was added into the high-risk Western-style diet with high-fat and low-vitamin D; in the current study, the selenium was added into a defined AIN-76A control diet.

With novel signaling pathways involving in carcinogenesis and angiogenesis have been discovered, whether preventive or therapeutic agents can target to these pathways are large unknown. For example, the polo-like kinase 3 (Plk3) has been recently found to phosphorylate and destabilize hypoxia-inducible factor 1α (HIF-1α). Plk3 can also phosphorylate and stabilize PTEN phosphatase, a known regulator of HIF-1α and tumor angiogenesis [32]. Whether the combination of selenium and sulindac can target on Plk3-HIF-1α-PTEN signaling, for colorectal cancer prevention and therapy, is under investigation. Recent reports have shown that gastrointestinal stromal tumors (GIST) account for approximately 1% to 3% of all malignant GI tumors [33], but the treatment for GIST is still a big challenge. Whether the combination of selenium and sulindac has similar effects on GIST prevention is unknown and needs to be investigated.

In conclusion, selenium and sulindac are synergistic to inhibit intestinal tumorigenesis in the *Apc/p21+/−* mice through impacting methylation, inducing the expression of p27, p53 and phosphorylation of JNK1, and suppressing β-catenin signaling. Current finding provides an important chemopreventive strategy using combination of anti-cancer agents instead of a single one. Therefore, the finding from this study has a great impact on cancer prevention and has a promising translational potential.

## Materials and methods

### Experimental animals and dietary supplementation

As described previously [3,7,31], the *Apc1638+/−* mice were mated with *p21−/−* mice to produce *Apc1638+/−/p21+/−* mice. At weaning (approximately 3–4 weeks), littermates were randomized to genetic/dietary groups and fed ad libitum AIN-76A diet,AIN-76A diet supplemented with 0.00004% (4.0 ppm) sodium selenite or the AIN-76A diet supplemented with 0.00004% (4.0 ppm) sodium selenite and 0.02% (200 ppm) sulindac (Research Diets Inc., New Jersey). The mice were weighed weekly and maintained on diet for 24 weeks or until they exhibited significant weight loss or other signs of extensive tumor formation. The animals were sacrificed by CO2 overdose, followed by cervical dislocation, and rapidly dissected for evaluation of tumors and fixation of tissues, as described previously [4,26,34]. This study was carried out in strict accordance with the recommendations in the Guide for the Care and Use of Laboratory Animals of the National Institutes of Health. The protocols were approved by the Animal Care Committee of the University of Illinois at Chicago (Protocol Numbers: 06–196 and 09–088).

### Mouse intestinal tumorigenesis analysis

The gastrointestinal tract from stomach through rectum was removed. The entire intestinal tract was opened longitudinally, and the stomach opened along the greater curvature to expose the mucosa. The full length of the intestinal tract was immediately examined for neoplastic lesions under a dissecting microscope. The location and the macroscopic features of all mucosal growth were recorded. Tumor incidence (percentage of mice with tumor), multiplicity (number of tumors per mouse), and size (tumor volume) were analyzed.

### Mouse intestinal epithelial cells collection and immunoblotting

Intestinal epithelial cells from each strain were isolated by incubating opened mouse intestine from duodenum as described recently [26,35]. Protein was extracted from the cell pellets isolated from the intestinal epithelial cells. Equal amounts of protein (30 μg) were size-fractionated on a 10% SDS gel and transferred onto a nitrocellulose membrane (Bio-Rad Laboratories, Hercules, CA) using a semi-dry transfer system (Bio-Rad). After blocking in 5% non-fat milk, blots were incubated with primary antibodies against p-JNK, p53, p27, Cyclin D1, CDK4 and β-catenin (Santa Cruz Biotechnology, Inc., Santa Cruz, CA), or β-actin (Sigma, St Louis, MO) for overnight at 4°C. Blots were washed with PBS containing 0.1% Tween, and incubated with secondary antibodies conjugated to HRP for 2 hours at room temperature. Immunoreactive proteins were visualized and analyzed. Immunoblotting intensities were quantified using Quanty One software (Bio-Rad, Hercules, CA).

### Methylation-specific polymerase chain reaction (MS-PCR)

Genomic DNA was isolated using a Dneasy Tissue Kit (Qiagen, Valencia, CA, USA) from the intestinal epithelial cells. Cytosine methylation was determined using EZ-DNA methylation kit (Zymo Research, Irvine, CA) according manufactory instruction. Briefly, under these conditions, unmethylated cytosine was converted to uracil, but 5’-methylcytosine remained as cytosine. The primers for methylation-specific PCR were designed using MethPrimer as reported previously [8]. The primers for the distal CpG cluster (100 bp, from −775 to −676) were: forward (−824 to −800) 5^′^-TGGTTTGAGAATTGGATTTAATTTT-3^′^, reverse (−641 to −665) 5^′^-TCCCAAAAAATCCCACTATATCTAA-3^′^, as this region was reported to play a critical role in the response to sulindac in our previous study [8]. The thermal cycler was programmed as following: denaturation at 95°C for 5 min, followed by 35 cycles of denaturation at 95°C for 45 s, annealing at 56°C for 45 s and extension at 72°C for 45 s, and then 72°C for 10 min at completion.

### Statistical analyses

Values for the average of intestinal tumor numbers were expressed as means ± SD. Analysis of variance was used to compare data derived from *Apc/p21* mice fed with AIN-76A control diet, AIN-76A plus sodium selenite, or AIN-76A plus sodium selenite and sulindac, to determine whether there was a significant effect of selenium alone or synergistic effect of selenium and sulindac on tumor prevention.

## Findings

Combination of selenium and sulindac significantly inhibited intestinal tumorigenesis, which was linked to induction of the expression of p27 and p53 and JNK1 phosphorylation, suppression of β-catenin signaling and demythelation on p21 promoter.

## Abbreviations

APC: Adenomatosis polyposis coli; p21: p21WAF1/cip1; MS-PCR: Methylation-specific polymerase chain reaction.

## Competing interests

All authors have no competing interests to declare.

## Authors’ contributions

YW designed and guided the experiments, BX, PN and DH conducted the experiments, BX and YW analyzed the data and wrote the manuscript. All authors read and approved the final manuscript.
